# Influence of the HIV GWG variant in the HIV infection progression in mono and HCV coinfected patients

**DOI:** 10.1097/MD.0000000000016376

**Published:** 2019-07-19

**Authors:** Flavia Hebeler-Barbosa, Viviam Milanez Massolini, Thais Watanabe, Giovanni Faria Silva, Alexandre Naime Barbosa, Rafael Plana Simões, Adriana Camargo Ferrasi, Paolo Marinho de Andrade Zanotto, Maria Inês de Moura Campos Pardini, Rejane Maria Tommasini Grotto

**Affiliations:** a São Paulo State University (Unesp), Medical School; b Molecular Biology Laboratory, Blood Transfusion Center; c São Paulo State University (Unesp), School of Agriculture, Botucatu; d Laboratory of Molecular Evolution and Bioinformatics (LEMB), Biomedical Sciences Institute, University of São Paulo (USP), São Paulo, Brazil.

**Keywords:** glicine-tryptophan-glicine variant, hepatitis C virus, human immunodeficiency virus sequence variability., human immunodeficiency virus subtype B, human immunodeficiency virus

## Abstract

The HIV subtype B is the most frequent in Brazil. The HIV subtype B’ codes the amino acids glicine-tryptophan-glicine (GWG) instead of glicine-proline-glicine on the tip of gp120 V3 loop. This variant was associated to a slower HIV progression in mono-infected patients; however, there is no information in coinfected patients. This study evaluated the infection progression of HIV variant B’ on the hepatitis C virus presence. RNA isolated from plasma of the 601 infected patients were used to human immunodeficiency virus (HIV) subtyping and to classify the virus according their syncytium-inducing ability. The HIV infection progression was evaluated by clinical and laboratorial data. The results showed a significant association between HIV B’ variant and CD4 count and time of AIDS in HIV mono-infected patients. Notwithstanding the fact that we did not find a direct association between GWG variant and AIDS and in HIV coinfected patients no mitigating effect due to GWG presence was found. We did observe that the association between GWG variant and CD4 counts is lost in coinfected patients. This is first work showing influence of the HIV GWG variant in coinfected patients. Nevertheless, the presence of the GWG variant can indicate a better prognostic in the mono-infected patients.

## 1 Introduction

### 1.1 Background

The human immunodeficiency virus (HIV) shows high genetic variability throughout its entire genome but the *env* gene is the most variable.^[1]^ This variability becomes particularly important in the HIV-1 gp120 V3 loop, in which the sequence variations have been associated with HIV antigenicity, immune dominance,^[2]^ and cytopathogenicity modifications.^[3]^

Using algorithms based on the position-specific scoring matrix (PSSM),^[4,5]^ the HIV gp120 V3 loop has been described as an important motif associated with HIV cytopathic activity. Non-syncytium-inducing (NSI) HIV has been described as less cytopathic and to be present in the asymptomatic phase. On the other hand, the syncytium-inducing (SI) variants appear during AIDS and present more cytopathic activity.^[6–9]^

The HIV major immunogenic region is the gp120 V3 loop,^[10,11]^ which has the sequence CTRPNNNTRKSIHI**GPG**RAFYTTGEII GDIRQAHC in most of the cases^[12]^ and exhibits a loop conformation due to presence of 2 cysteine residues at the end of sequence that interact by a disulfide bond. The tip of the V3 loop presents the conserved motif, a glicine-proline-glicine (GPG), in the HIV subtype B, which is the most frequent in Brazil. Nevertheless, a HIV-1 subtype B variant (named B’) has been described in Brazil, in which the proline is substituted for a tryptophan in the V3 loop tip, creating a glicine-tryptophan-glicine (GWG) motif.^[13–15]^

Previous studies have demonstrated the association between the GWG variant and progression of HIV infection in mono-infected patients.^[16–20]^ Importantly, the patients with the GWG variant present slower HIV infection progression and better prognosis. However, so far there are no reports concerning the association of the GWG HIV variant and infection evolution in HIV patients coinfected with hepatitis C virus (HCV).

On the other hand, particular sets of human genetic polymorphisms have been found to differ in patients coinfected with HIV and HCV when compared to patients mono-infected with HIV or HCV.^[21,22]^ Likewise, the association of the host genetic polymorphisms with the progression of hepatic fibrosis is different in HCV mono-infected patients compared to HIV/HCV coinfected patients.^[23]^

The coinfection of HIV/HCV has been described in 5% to 25% of patients with HIV^[24,25]^ and HIV/HBV coinfection in approximately 10% of HIV patients.^[26,27]^ The coinfection of HIV/HCV leads to a faster immunological debilitation.^[28]^

Considering that the tip of V3 loop is the major immunodominant region of HIV gp120,^[29]^ the presence of the GWG motif could modify the immunological status of HIV mono-infected or HIV/HCV coinfected patients.

Immune debilitation in HIV/HCV coinfection has been described as a consequence of the hepatic cells signaling pathway that occurs when HIV gp120 interacts with CD4 and C-C chemokine receptor type 5 (CCR5) or CXCR4 receptors and coreceptors.^[30]^ One way of the immune debilitation that occurs in HIV/HCV coinfection could be mediated by the inhibition in interferon function. Type I interferon have been described as essential molecules with antiviral action^[31,32]^ and the presence of HCV could alter cell signaling controlling interferon secretion.^[33]^

### 1.2 Objectives

Considering the association of the HIV variant B’ (GWG) and a slower progression of HIV infection in mono-infected patients, the goal of this study was to evaluate the presence of HIV variant B’ and its association with infection progression in patients’ coinfected with HIV/HCV.

## 2 Methods

### 2.1 Study design, setting, and participants

Aliquots of Ethylenediamine tetraacetic acid-anticoagulated peripheral blood were collected from 601 HIV patients from the health service of the Botucatu region in Sao Paulo, Brazil. Inclusion criteria were that patients must be 18 years old or more and be infected by HIV subtype B confirmed by molecular assays. Exclusion criteria included the presence of other hepatic diseases, patients coinfected with more than 2 virus and pregnant women.

The patients were divided into 2 groups: group 1 (G1) included the HIV mono-infected patients and group 2 (G2) included the HIV/HCV coinfected patients. The patients included in study are followed in health service after first diagnosis of the HIV or HCV infection. HIV/HCV coinfection was defined as individuals with positive serology for both viruses and confirmed diagnosis by molecular tests. HIV-1 and HCV RNA levels were measured using the Abbott Real Time HIV-1 assay and Abbott Real Time HCV assay, respectively. Within of each group patients were grouped according the HIV gp120 V3 loop tip motif (GWG or GPG).

### 2.2 Variables, data measurement, bias, study size

RNA was isolated from plasma using the QIAamp RNA Viral mini kit (Qiagen, Valencia, CA). RNA was used to amplify the HIV C2–C3 genomic region by nested reverse transcription polymerase chain reaction. The reactions were performed using Platinum Taq DNA Polymerase (Life Technologies, CA) according to the manufacturer's specifications using primers described by Delwart et al.^[34]^ For the first PCR, primers ED5 (5′-ATGGGATCAAAGCCTAAAGCCATGTG-3’; positions 6556–6581) and ED12 (5′-AGTGCTTCCTGCTGCTCCCAAGAACCCAAG-3’; positions 7822–7792), were used to amplify the 1.2-kb variable (V1) through V5 coding domains of the surface protein. For the nested PCR, primers ED31 (5′-CCTCAGCCATTACACAGGCCTGTCCAAAG-3’; positions 6816–6844) and ED33 (5′-TTACAGTAGAAAAATTCCCCTC-3’; positions 7359–7380)^[34]^ were used to amplify the 0.5-kb C2 through C3 coding domains of surface protein. The PCR products were sequenced using Big Dye Cycle Sequencing, version 3.1 (Life Technologies), and the sequencing runs were conducted in a Genetic Analyzer 3500 (Life Technologies) according to the manufacturer's specifications with the same primers used for the nested PCR reactions.

Sequences reads were evaluated using Phred^[35,36]^ with a quality score of 20. Sequences were analyzed in the BioEdit program (www.mbio.ncsu.edu/BioEdit/bioedit.html) to obtain the amino acid sequence. The amino acid sequences obtained were used to define the tip of the V3 loop (GPG, GWG) and to make inferences about the HIV syncytium inducing (SI) ability using the Web PSSM available at the https://indra.mullins.microbiol.washington.edu/webpssm/.^[4,5,37]^

HIV infection progression was evaluated by clinical (i.e., AIDS presence, HIV risk factors) and laboratory data (i.e., CD4 count, plasma viral load, time of AIDS by HIV, cytopathic ability) obtained from the patient's medical records.

AIDS was defined to Centers for Disease Control and Prevention (CDC) B and C stages, according CDC classification criteria and HIV risk factors were obtained from medical records. CD4 count and plasma viral load was obtained at collection date (moment which patient was included in the study). Time of AIDS was obtained from patient's medical records (time of AIDS was defined as time between AIDS diagnosis and the patient's inclusion in the study).

### 2.3 Data analysis and statistical methods

Statistical analyses were performed to evaluate the significant differences that could be associated with the progression of HIV infection. Analyses were carried out between GWG and GPG variants within of each group. Time of AIDS, cytopathic ability (NSI or SI), AIDS presence, HIV risk factors, CD4 count, and HIV plasma viral load were analyzed. We also assessed qualitative data on cytopathic ability (NSI or SI), AIDS presence, HIV risk factors, and the Pearson Chi-squared test was applied to check if the attributes in a contingency table were statistically independent.

For the quantitative data (time of AIDS, CD4 count, and HIV plasma viral load), the Wilcoxon nonparametric test for independent samples was applied to determine if the differences between the means were significantly different. Time of AIDS for patients without AIDS was considered equal to zero.

The Kaplan–Meier and Cox proportional survival analyses were performed to evaluate the influence of the GWG and GPG motifs on the patient's immunological status (endpoint: CD4 count more than 200 cell/mm^3^).^[38–40]^

The level of significance for all statistical tests was set at 0.05.

### 2.4 Ethics approval and consent to participate

This study was approved by the Research Ethics Committee of Botucatu Medical School, UNESP (document number 1.258.824). The written informed consent was obtained from all patients. The study including clinical data collection questionnaires obtained from physician.

## 3 Results

### 3.1 Participants

From the 601 patients included in this study, 87 were excluded because they were infected by an HIV subtype other than B (exclusion criteria).

### 3.2 Descriptive data, outcome data, and main results

**Figure 1:**
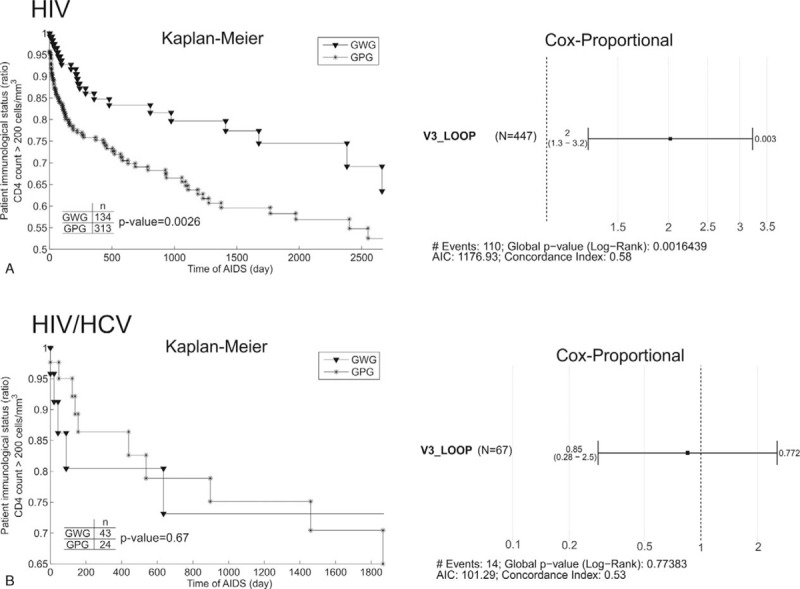
The Kaplan–Meier and Cox proportional analyses of the GWG and GPG motifs influence on patient immunological status for: (A) HIV mono-infected and (B) HIV/HCV coinfected patients. The HIV mono-infected patients with GWG motif in tip of V3 loop showed higher CD4 levels by the time according both: Kaplan–Meier and Cox proportional analyses. GPG = glicine-proline-glicine, GWG = glicine-tryptophan-glicine, HCV = hepatitis C virus, HIV = human immunodeficiency virus.

The characteristics of the 514 patients with HIV subtype B that were included in the data analysis were shown in Table 1. The statistical analysis done within groups had no significant difference (*P* > .05) in frequencies of the GWG or GPG motif according to cytopathic ability, AIDS presence and HIV risk factors for both HIV mono-infected and patients coinfected with HIV/HCV (Table 2). Nevertheless, there were significant associations between GWG variant and the CD4 count (*P* < .05) and time of AIDS (*P* < .05) in HIV mono-infected patients. Interestingly, there was no difference among these variables in the HIV/HCV coinfected groups (Table 3). The Kaplan–Meier and Cox proportional survival analyses indicated a higher CD4 level over time for the HIV mono-infected patients with the GWG variant (Fig. 1A). This behavior was not observed in HCV/HIV coinfection (Fig. 1B).

**Table 1:**
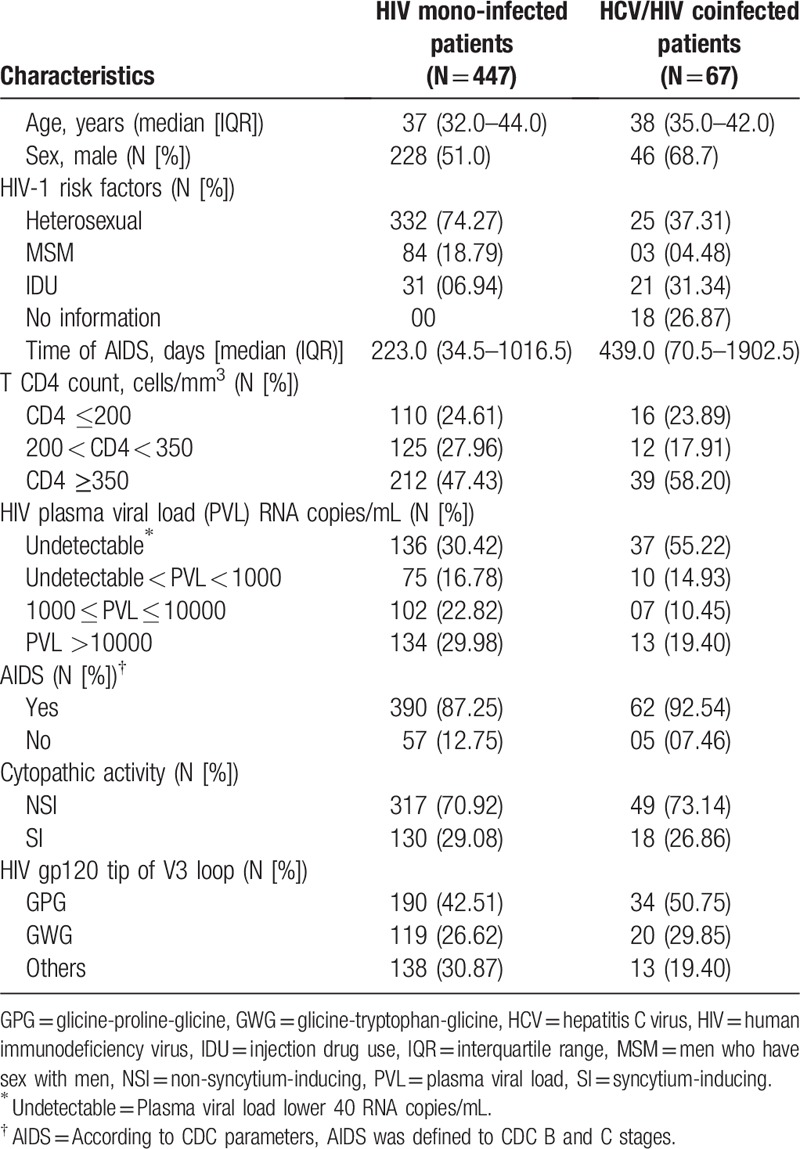
Demographical, virological, immunological, and clinical characteristics of the patients included in this study (n = 514).

**Table 2:**
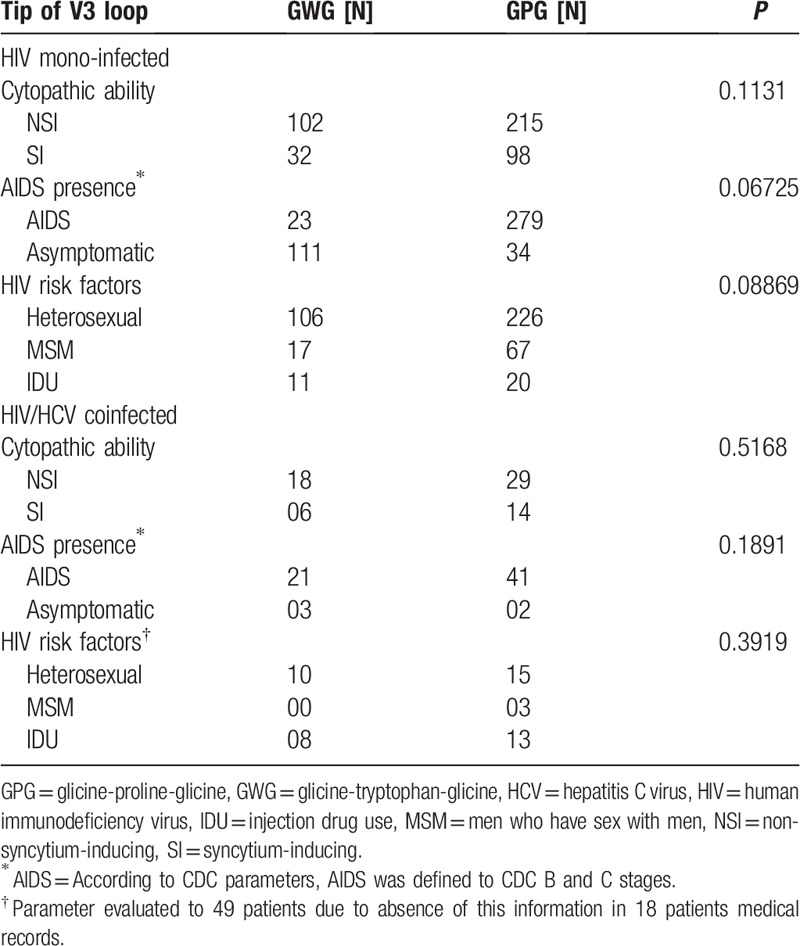
Frequencies for glicine-tryptophan-glicine or glicine-proline-glicine motif according clinical or laboratory data. The *P*-values were obtained from Pearson Chi-squared test.

**Table 3:**
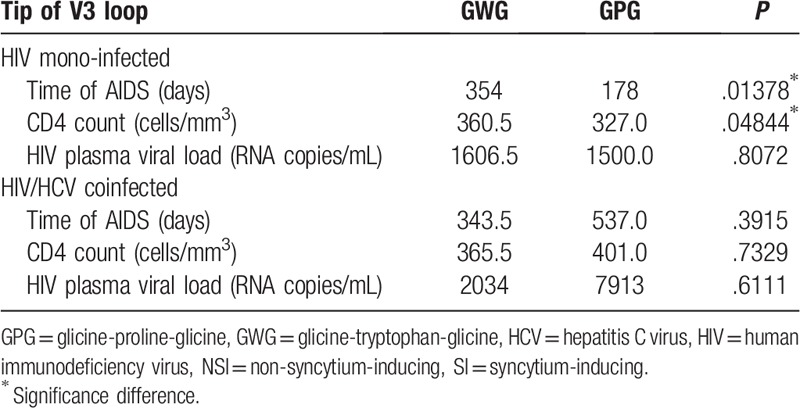
Glicine-tryptophan-glicine or glicine-proline-glicine motif according time of time of acquired immune deficiency, CD4 count, and human immunodeficiency virus viral load. The *P*-values were obtained from Wilcoxon nonparametric test for independent samples.

**Figure 2:**
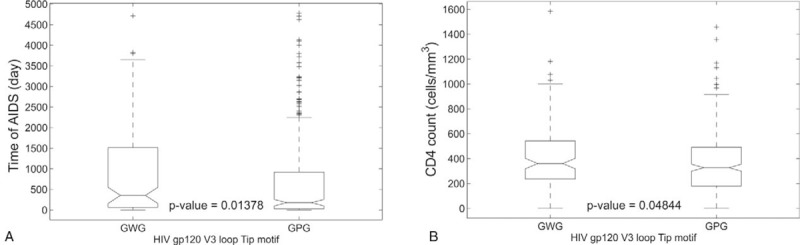
Boxplot of the data distribution to: (A) time of AIDS and (B) CD4 count according HIV gp120 V3 loop tip (GPG or GWG). The *P*-values were obtained from Wilcoxon nonparametric test for independent samples. GPG = glicine-proline-glicine, GWG = glicine-tryptophan-glicine.

Box plots of the significant data were plotted to illustrate the differences between the means. The HIV GWG variant was associated with higher CD4 levels and time of AIDS (Fig. 2).

## 4 Discussion

Although several HIV subtypes have been described in the world,^[41]^ subtype B is the major subtype circulating in America,^[42]^ including Brazil.^[15]^ This HIV subtype contains the GPG motif in the tip of the V3 loop. The HIV subtype B variant B’ has been reported in Brazil, which shows the GWG motif in the tip of the V3 loop.^[17]^

In this study, the GWG (B’ variant) was found in 30.0% and 35.8% of the HIV mono-infected patients and HIV/HCV coinfected patients, respectively. Despite the fact that the presence of this variant in the coinfected patients is poorly understood, the studies in Brazil with HIV mono-infected patients have shown different proportions of the GWG variant. In this study, the frequency of the GWG variant (Table 1) is in agreement with recent reports from Southeast Brazil.^[20,43]^

There is evidence in the literature concerning the association between HIV GWG variant and slower disease progression in HIV mono-infected patients.^[17–20,44]^ In this work, nevertheless we did not establish the direct association between the GWG variant and AIDS presence, cytopathic ability, or HIV risk factors in the mono-infected group (Table 2). However, it may be only a consequence of characteristics of the patients included in this study, in which aids presence was predominant (Table 1).

Crucially, as the most important result of this work we show here that the GWG variant was associated with higher levels of CD4 in HIV mono-infected patients (Table 3 and Fig. 1). The CD4 count has been already described as the most important prognostic marker of progression during HIV infection.^[17]^ Furthermore, the time of AIDS was longer in the HIV mono-infected patients with the GWG motif at the tip of the V3 loop (Fig. 2), suggesting that the GWG motif can be a marker to more time living with the disease.

The V3 loop is the major HIV immunogenic region.^[45]^ The GPG motif has been associated with the β-hairpin conformation of the V3 loop,^[46]^ which is essential for the interaction between HIV and CCR5 and/or CXCR4, which are HIV entry coreceptors.^[47]^ In this way, the change of a proline for a tryptophan in the tip of the V3 loop (GPG GWG) may result in conformational modifications that could influence the immune response to the virus,^[13]^ leading to an immunological protective effect for HIV patients who harbor the B’ variant (GWG motif). In addition, the presence of the GWG motif could modify the interaction with the HIV coreceptor, leading to lower progression of HIV infection and occurrence of opportunistic infections.

On the other hand, the levels of CD4 and the time of AIDS were the same in coinfected patients, independent of the presence of the GPG or GWG motif (Fig. 1). These results represent the first report about the GWG variant in HIV/HCV coinfected patients.

We did observe that in HIV/HCV coinfected patients the protective effect provided by the GWG variant is lost in the presence of HCV. Despite the fact that the mechanism involved in this process is still unknown, we could suppose that the immunological response changed due to the presence of HCV.

Some mechanisms may agree with this hypothesis. It was shown that HIV/HCV coinfection results in immune debilitation due to hepatic cell signaling pathways that are triggered when the HIV gp120 interacts with CD4.^[30]^ Moreover, the presence of HCV interferes in signaling pathways involved in type I interferon release,^[33]^ which leads to antiviral effects.^[31,32]^ The presence of HCV also enhances HIV replication.^[48]^ The CD4 T depletion has shown to have a profibrogenic role, leading the fibrosis progression in HCV infection.^[49]^

One could argue that, the HCV coinfection could increase HIV replication,^[48]^ leading to increased CD4 depletion and a weakened immune response.^[49]^ This effect could also be associated with HCV-dependent type I interferon that would impair infection control by also, intensifying the immunological debilitation.^[33]^

In this way, the HCV presence can explain the lack of protective effect of the GWG variant in coinfected patients. New studies should be conducted to investigate the mechanisms contributing to this process.

This is first study that evaluated the presence of the HIV GWG variant in coinfected patients. The presence of the GWG variant in HIV mono-infected patients can indicate a better prognostic in the clinical practice. Nevertheless, the HIV GWG variant did not influence the decline of the CD4 counts in coinfected patients.

## Acknowledgments

We thank all patients that participated of this study.

## Author contributions

**Conceptualization:** Maria Inês de M.C. Pardini, Rejane Maria Tommasini Grotto.

**Data curation:** Flavia Hebeler-Barbosa, Viviam Milanez Massolini, Thais Watanabe, Giovanni Faria Silva, Alexandre Naime Barbosa, Adriana Camargo Ferrasi.

**Formal analysis:** Rafael Plana Simões.

**Funding acquisition:** Paolo Marinho de Andrade Zanotto, Rejane Maria Tommasini Grotto.

**Investigation:** Flavia Hebeler-Barbosa, Viviam Milanez Massolini, Giovanni Faria Silva, Alexandre Naime Barbosa, Rafael Plana Simões, Paolo Marinho de Andrade Zanotto, Rejane Maria Tommasini Grotto.

**Methodology:** Flavia Hebeler-Barbosa, Viviam Milanez Massolini, Thais Watanabe, Giovanni Faria Silva, Alexandre Naime Barbosa, Adriana Camargo Ferrasi.

**Project administration:** Rejane Maria Tommasini Grotto.

**Resources:** Rejane Maria Tommasini Grotto, Maria Inês de M.C. Pardini, Paolo Marinho de Andrade Zanotto.

**Supervision:** Paolo Marinho de Andrade Zanotto, Rejane Maria Tommasini Grotto.

**Writing – original draft:** Flavia Hebeler-Barbosa, Rafael Plana Simões, Maria Inês de M.C. Pardini, Rejane Maria Tommasini Grotto.

**Writing – review & editing:** Flavia Hebeler-Barbosa, Rafael Plana Simões, Maria Inês de M.C. Pardini, Paolo Marinho de Andrade Zanotto.

Flavia Hebeler-Barbosa orcid: 0000-0001-7355-5179.
